# ALK Rearrangement–Positive Pancreatic Cancer with Brain Metastasis Has Remarkable Response to ALK Inhibitors: A Case Report

**DOI:** 10.3389/fonc.2021.724815

**Published:** 2021-09-06

**Authors:** Kai Ou, Xiu Liu, Weihua Li, Yi Yang, Jianming Ying, Lin Yang

**Affiliations:** ^1^Department of Medical Oncology, National Cancer Center/National Clinical Research Center for Cancer/Cancer Hospital, Chinese Academy of Medical Sciences and Peking Union Medical College, Beijing, China; ^2^Department of Pathology, National Cancer Center/National Clinical Research Center for Cancer/Cancer Hospital, Chinese Academy of Medical Sciences and Peking Union Medical College, Beijing, China; ^3^Department of Interventional Therapy, National Cancer Center/National Clinical Research Center for Cancer/Cancer Hospital, Chinese Academy of Medical Sciences and Peking Union Medical College, Beijing, China; ^4^Key Laboratory of Gene Editing Screening and Research and Development (R&D) of Digestive System Tumor Drugs, Chinese Academy of Medical Sciences and Peking Union Medical College, Beijing, China

**Keywords:** pancreatic cancer, brain metastases, ALK Kinase, EML4-ALK fusion protein, crizotinib, alectinib

## Abstract

Patients with metastatic pancreatic cancer typically have poor prognosis due to the limited effectiveness of existing treatment options. ALK rearrangement–positive is rare in pancreatic cancer, but may occur in those with KRAS-wild type. We present a 34-year-old young man with ALK rearrangement–positive and KRAS-wild pancreatic cancer who had a remarkable response to crizotinib after resistance to prior chemotherapy and re-response to alectinib after brain metastases developed. This clinical observation suggests that comprehensive molecular profiling to guide targeted therapies is not only feasible, but also significantly improves survival outcomes for a subgroup of patients with pancreatic cancer.

## Introduction

According to the estimation of Global Cancer Statistics 2020, pancreatic cancer ranks seventh among cancer-related deaths worldwide (1). The median overall survival time (mOS) for patients with locally advanced pancreatic cancer is 8-12 months, while that is only 3-6 months for patients with distant metastases. Although surgery is the only way to cure pancreatic cancer, about 80% of patients’ tumors are unresectable at the first visit (2). Chemotherapy is the primary means of treatment for most patients. Unfortunately, existing chemotherapy regimens have limited effectiveness. First-line combination chemotherapy, for instance, using gemcitabine plus nab-paclitaxel (GN) or FOLFIRINOX (5- fluorouracil, folinic acid, oxaliplatin, and irinotecan) only increases the mOS of patients with advanced pancreatic cancer by 2-4 months in phase III trials (2, 3). In the cases of disease progression after GN therapy, nanoliposomal irinotecan (nal-IRI) can be given as second-line therapy, according to the landmark phase III NAPOLI-1 trial (4). However, therapeutic options are very limited after the failure of the standard treatment.

The development of molecular biology has promoted targeted therapy and immunotherapy based on specific biomarkers and has resulted in benefits in the outcomes of several tumors. KRAS mutation is the most common alterations in pancreatic cancer, the frequency is over 90% (5–8) and appear to occur in the early stage of tumor, while inactivated tumor suppressor genes p16/CDKN2A, TP53, and SMAD4 occur in over 50% patients (7–9). Although these common genetic variations play a role in early detection or prognosis prediction to some extent, they have not yet matched more effective treatments. DNA mismatch repair gene inactivation occurs in approximately 2 to 3% patients (5, 6, 8–10) and is most commonly seen in the familial pancreatic cancer. Besides, up to 17% of patients harbor BRCA2 or PALB2 mutations in the familial setting. Progress has been made in the clinical study of a number of drugs aimed at these patients (11–14). DNA mismatch repair deficient results in an increased sensitivity to immunotherapy (11–13). Olaparib maintenance therapy has been shown to be effective in patients with metastatic pancreatic cancer with BRCA mutations (14). TRK inhibitors have been approved for tumors that harbor ROS1, NTRK1, NTRK2, and NTRK3 gene fusions.

ALK gene is located on chromosome 2p23 and is physiologically expressed in fetal neural cells (15). ALK rearrangements with various partner genes result in ALK fusion proteins and constitutive ALK activation. ALK rearrangement was reported in anaplastic large cell lymphoma in 1994 for the first time (16) and have been characterized in multiple solid tumors, including esophageal squamous cell carcinoma (17), thyroid, breast, colorectal cancers (18, 19) and non–small-cell lung cancer (NSCLC) (20). Patients with ALK rearrangement–positive have a better prognosis than negatives in multiple tumors such as anaplastic large cell lymphoma (17, 21)and inflammatory myofibroblastic tumors (IMTs) (22). ALK fusion proteins have become an attractive target of precision medicine. ALK inhibitors, including crizotinib, ceritinib, alectinib and lorlatinib have proven efficacy in patients with ALK rearrangement–positive tumors.

ALK rearrangements were first reported in pancreatic cancer in 2017 and only 7 occurrences (23) have been identified to date. In a study of over 3,100 patients with pancreatic cancer, 5 patients had ALK rearrangements, and none of them had KRAS mutations.

Here, we present the eighth case of ALK rearrangement–positive in metastatic pancreatic cancer who initially had a response to crizotinib, then experienced progression with new development of brain metastasis, which responded to alectinib.

## Case

A 34-year-old male presented with persistent pain in the upper abdomen was admitted to our hospital on June 14, 2019. He had no back pain, vomiting, jaundice, weight loss, loss of appetite. He had a history of splenectomy because of a traffic accident and appendectomy because of appendicitis. He had no history of diabetes, chronic pancreatitis, obesity, and no family history of cancer. He had a long history of heavy smoking (approximately 24 years*15 cigarettes/day) and alcohol consumption (approximately 22 years*100g/day). Physical examination revealed multiple palpable left axillary lymph nodes with 2cm*2cm in maximal size.

**Figure 1:**
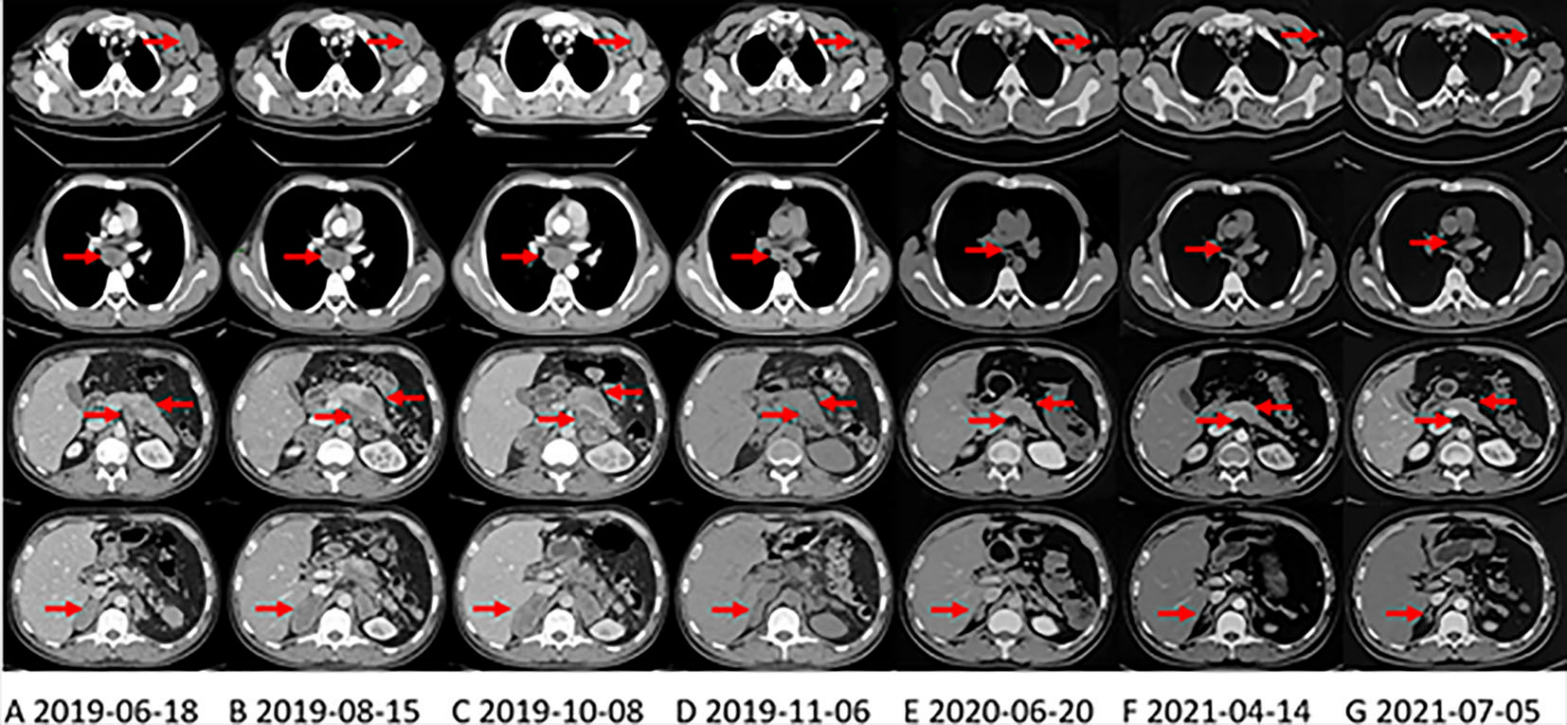
CT shows the changes of primary pancreatic lesion and metastases in the right axillary, mediastinum, and left adrenal gland with treatment. The arrows in each layer from top to bottom refer to the left axillary lymph node, mediastinal lymph node, pancreas and abdominal lymph nodes, and the right adrenal gland. **(A)** CT at the first evaluation of the patient. **(B)** CT evaluation of progressive disease (PD) after first-line irinotecan, oxaliplatin and capecitabine (XELOXIRI) treatment for 3 cycles. **(C)** CT evaluation of PD after second-line paclitaxel and gemcitabine treatment for 2 cycles. **(D)** third-line Crizotinib treatment One month later, the patient’s tumor shrank significantly, and CT was evaluated as partial response (PR). **(E)** when the patient was initially found to have brain metastases, the extracranial lesions were still stable, and the patient continued to take Crizotinib. **(F)** On April 13, 2021, The extracranial lesions are stable and the patient has been receiving Alectinib since June 2020. **(G)** at the latest CT evaluation, the extracranial lesions are still stable.

Abdominal contrast-enhanced CT scan revealed a 10.0*4.3-cm mass on the pancreatic body and tail with blurred borders with bilateral adrenal multiple metastases and multiple swollen lymph nodes in the mediastinum, left axilla, abdominal cavity, retroperitoneum and mesentery (**Figure 1A**). There are no definite masses in either lung. His serum CA 19-9 level was elevated at 219.6 U/mL (normal <33 U/mL).

Left axillary lymph node biopsy was performed on July 3, 2019 and the pathological examination revealed poorly differentiated adenocarcinoma infiltration in fibrous tissue. Immunohistochemical (IHC) staining indicated that the cells were positive for CA19-9, CK18, CK19, CK7, AE1/AE3, MUC5AC, and negative for CDX-2, CK20 and MUC6. Ki-67 showed a 10% proliferative rate. For the sake of reducing patients’ damage, we have not obtained the results of the patient’s primary tumor through puncture and other methods. However, the patient had upper abdominal pain, a mass in the pancreatic area with swelling of peripheral lymph nodes on imaging, and low enhancement with uneven enhancement on enhanced CT, which were consistent with the manifestations of pancreatic cancer. The puncture pathological section of the patient’s lymph node metastasis showed adenocarcinoma. The results of immunohistochemical marker detection and the elevation of tumor markers CEA and CA19-9 all indicate that the tumor originated from the digestive system. Based on the above information, the tumor is in line with pancreatic cancer.

First-line chemotherapy with XELOXIRI (irinotecan 260mg d1, oxaliplatin 140mg d2, and capecitabine 1.0g in the morning and 1.5g in the evening po. d1-d7)every two weeks was administered from July 5, 2019. The toxicities were acceptable. However, after 3 cycles of treatment, CT performed on August 15, 2019 showed that the primary tumor and metastases enlarged significantly (**Figure 1B**). His serum CA 19-9 level was also elevated at 477.8 U/ml. Then, second-line chemotherapy with nab-paclitaxel 200mg d1 & d8 and gemcitabine 1600mg d1 & d8 every three weeks was administered for 2 cycles from August 22, 2019, the patient developed grade 1 alopecia (according to NCI-CTC v.5.0). However, the symptoms of abdominal pain worsened and the tumors progressed significantly (CT scan on October 8, 2019, **Figure 1C**) and serum CA19-9 increased slightly to 449.90 u/ml.

Subsequently, next-generation sequencing (NGS) (panel of 520 cancer-related genes) was performed on the tumor tissue from left axillary lymph node. There were no mutations in EGFR, KRAS, BRAF. The tumor mutation burden (TMB) was 0.8 mutations/Mb and microsatellite status were stable (MSS). However, primary/reciprocal ALK fusion (EML4(exon 6)–ALK (exon 20)/ALK (exon 20)-EML4(exon 17)) was detected. Other genetic changes include ARID1B (c.821_823delCCG, 5.34%) and KEAP1(c.425C>T, 8.40%). ALK fusion was further confirmed by Fluorescence *in situ* hybridization test (FISH) and immunohistochemistry (IHC) (**Supplementary Figure 1** A H&E; B IHC; C FISH; D NGS).

**Figure 2:**
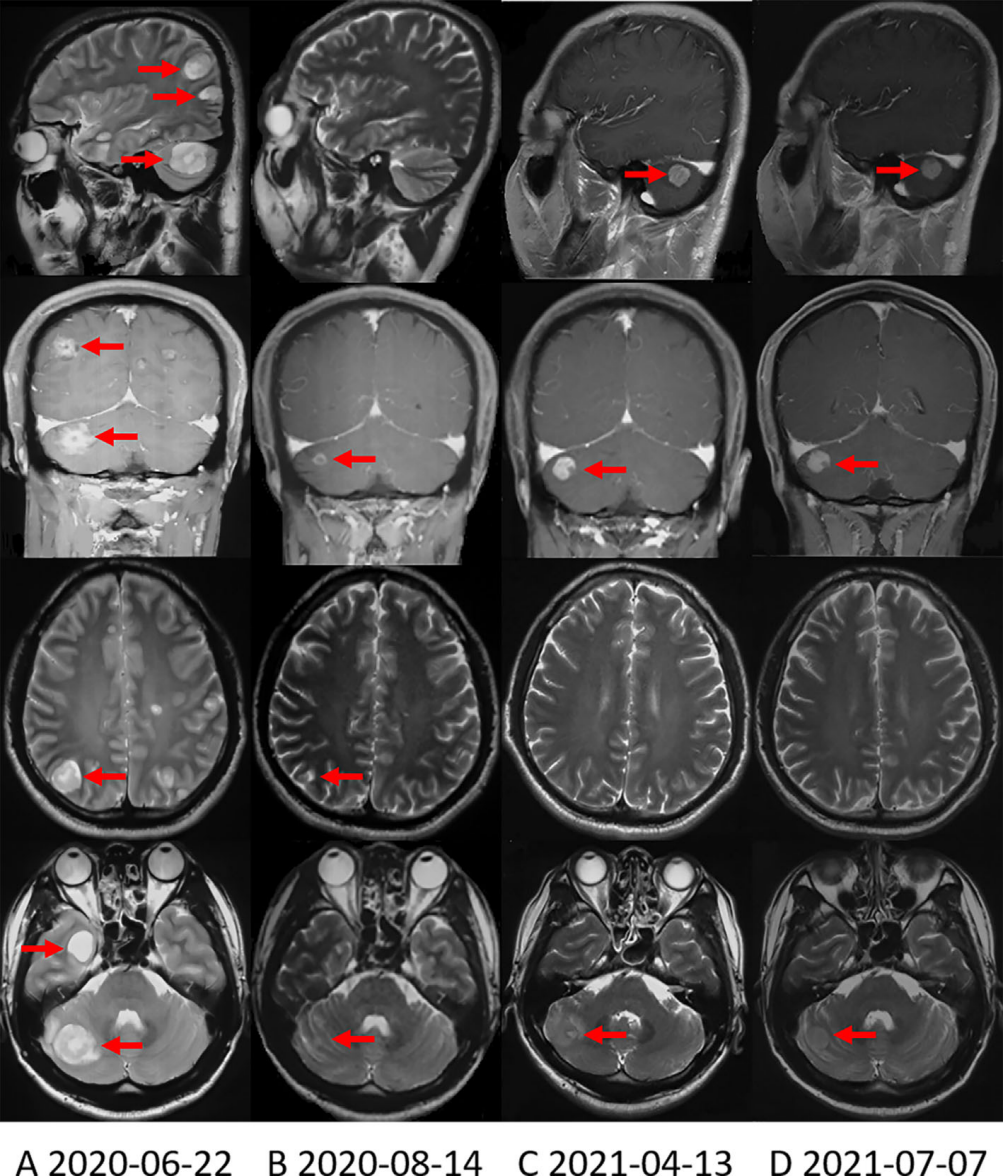
Brain MRI shows the change in brain metastases with treatment. **(A)** Brain MRI when the brain metastases first appeared. **(B)** Brain MRI showed that the brain metastases were significantly reduced after applying alectinib for nearly 2 months. **(C)** On April 13, 2021, Brain MRI showed that the right cerebellum lesion increased in size. **(D)** On July 7, 2021, the most recent Brain MRI showed that the brain metastases were stable.

Since October 14, 2019, the patient received 250mg of crizotinib twice daily. Apparent clinical remission was achieved. The palpable left axillary lymph nodes shrank quickly after 3 days of treatment. The patient’s abdominal pain disappeared and CT showed the tumor shrank significantly after 1 month (**Figure 1D**). The adverse event was grade 1 diarrhea. The treatment lasted for 8 months. On June, 2020, the patient complained of headache, decreased vision in the left eye and disturbance of balance in the left limb, CT and MRI on June 22, 2020 showed a progression in brain metastasis (**Figure 2A**). Interestingly, the extracranial lesions (**Figure 1E**) and serum CA19-9 remained durable responses. Then, Alectinib 600mg twice a day was administered from 23 June, 2020. The above symptoms relieved rapidly and no adverse events occurred during the treatment. The brain lesions shrank significantly (MRI scan on August 14, 2020, **Figure 2B**). However, after continuing the treatment for nearly 10 months, on April 13, 2021, the patient’s reexamination of the brain MRI showed that the right cerebellum (**Figure 2C**) and left frontal lobe lesions increased in size. The brain metastases were progressing, but the extracranial lesions were still stable (**Figure 1F**). The patient underwent a circulating tumor DNA (ctDNA) test on April 26, 2021. As a result, 10 gene mutations were found: AMER1 p.S794*, ATR p.E2626*, CCND2 p.A109D, CHEK1 p.S193Y, CREBBP p.S1599R, ERCC4 p.T660K, IL7R p.H279N, MRE11A p.A147D, SETD2 p.R87M, SETD2 p.M1080_E1081delinsI*, ctDNA testing has not yet prompted the basis for medication. Therefore, on the basis of continuing the application of alectinib, the patient received brain radiotherapy from June 1 to July 3, 2021. In the latest examination, brain lesions (**Figure 2D**) and other parts (**Figure 1G**) of the disease are stable, and the patient is currently being treated with alectinib.

**Figure 3:**
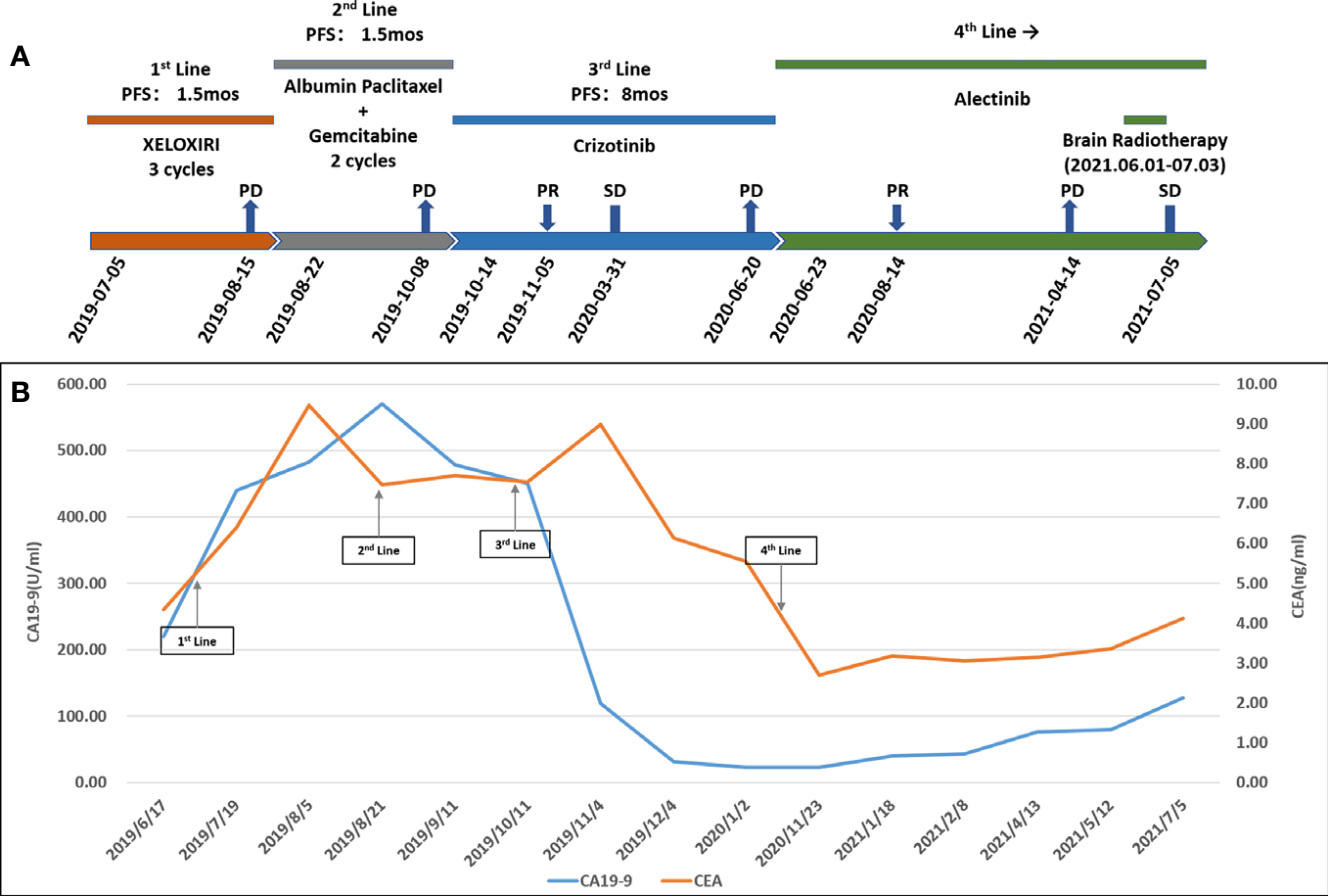
**(A)** Treatment process. PFS, progression-free survival; XELOXIRI, irinotecan, oxaliplatin and capecitabine; PD, progressive disease; SD, stable disease; PR, partial response; mos, months. **(B)** Change in cancer antigen 19-9 (CA19-9) (U/ml) levels and carcinoembryonic antigen (CEA) (ng/ml) levels with treatment.

The patient’s treatment process and changes in tumor markers are shown in **Figures 3A**, **B**, respectively. Due to COVID-19 epidemic, the patient’s tumor markers from January 2020 to November 2020 haven’t been successfully collected.

## Discussion

To the best of our knowledge, only eight cases with ALK rearrangement–positive pancreatic cancers have been reported to date, including the present one (**Table 1**). There were three females and five males. The average and median age of onset was 42 and 38 years old (range 32-72), respectively. Except for a patient aged 72, the age of onset of all other patients were under 50 years old. The main body of the original pancreatic lesions was in the head of the pancreas (7/8), except in this case (located in the body and tail of the pancreas). 4/5 of the patients were diagnosed as metastatic disease, and 1/5 of the patient’s disease progressed after classic pancreaticoduodenectomy (Whipple resection) post neoadjuvant chemotherapy and stereotactic body radiation therapy (SBRT). The metastatic site included distal lymph nodes (2/4), liver (2/4), ovaries (1/4), peritoneum (1/4), and adrenal glands (1/4).

**Table 1:** Characteristics of all eight cases with ALK-positive pancreatic cancer which reported to date.

case	sex	Age(yr)	stage at diagnosis	primary tumor location	distant metastatic site	ALK Rearrangement	other molecular alterations (MAF/CN)	ALK inhibitor	best overall response	duration of survival (months)
**1**	M	35	locally advanced: borderline resectable	head	retroperitoneal (left periaortic and retrocaval) and posterior mediastinal lymph nodes	Exon 13 EML4-exon 20 ALK	BAP1 c.1714delC(7%); NFE2L2 c.40C>T (5%); MCL1 amplification (7)	1. Crizotinib; 2. Ceritinib; 3. Alectinib	SD	52^a^
**2**	F	32	metastatic	head	liver; ovaries; peritoneum	Exon 6 EML4-exon 20 ALK	CDKN2A/B homozygous deletion; MYC amplification (82); TP53 c.325_326insTTCG (59%)	Crizotinib	PD	20
**3**	M	34	locally advanced: unresectable	head	None	Exon 3 STRN-exon 20 ALK	None	Crizotinib+ external-beam; RT+ gemcitabine	SD	10^a^
**4**	M	46	UK	head	UK	Exon 6 EML4-exon 20 ALK	CDKN2A homozygous deletion; TP53 c.994-1G>C (51%); SMAD4 c.247C>T (51%); FGFR1 c.422C>G (65%)	1. Crizotinib; 2. Alectinib	PD	5^a^
**5**	M	43	UK	UK	UK	Exon 6 EML4-exon 20 ALK	STK11 c.226delG (7%)	UK	UK	UK
**6**	F	72	UK	head	UK	Exon 12 DCTN1-exon 20 ALK	GNAS (R201H); TP53 (R432X); CDKN2A (R80X)	None	UK	UK
**7**	F	41	metastatic	uncinate process	liver	PPFIB P1-ALK	NF1 and TP53 inactivating mutations; CDKN2A/B loss; BRCA2 c.8007A>G (silent); CDKN1B 407A>G	1. Alectinib; 2. lorlatinib	PR	10^a^
**8^b^**	M	34	metastatic	body and tail	brain; mediastinum, left axilla, abdominal cavity, retroperitoneum and mesentery lymph nodes; bilateral adrenal;	Exon 6 EML4-exon 20 ALK	Exon 20 ALK- exon 17 EML4; ARID1B c.821_823delCCG (5.34%); KEAP1 c.425C>T (8.40%)	1. Crizotinib; 2. Alectinib	SD	36^a^

a, at the time of report, patient was still alive; b, our case; PD, progressive disease; PR, partial response; SD, stable disease; UK, unknown.

All of the eight cases were KRAS wild type. According to the real-time targeted genomic profile analysis of 3594 pancreatic ductal adenocarcinomas (PDAC) patients published by Singhi AD et al, KRAS alterations were absent in 12% PDACs (n=445). BRAF alterations were the most prevalent in this cohort (n=47, 11%), which also harbored kinase fusions including ALK (n=5, 1.1%), FGFR2 (n=12, 2.7%), RAF (n=7, 1.6%), RET (n=4, 0.9%), MET (n=2, 0.4%), NTRK1(n=2, 0.4%), ERBB4 (n=1, n=0.2%), and FGFR3(n=1, n=0.2%), etc. These fusions were mutually exclusive and were not present in PDACs with KRAS alterations. No other kinase fusions were reported in the published 7 cases. But in the present case, two types of gene fusion, EML4(exon 6)–ALK (exon 20) and ALK (exon 20)-EML4(exon 17) were identified.

In our dataset, the most common type of ALK rearrangement was EML4-ALK (5/8), other types of fusion include STRN-ALK (1/8), DCTN1 –ALK (1/8) and PPFIBP1-ALK (1/8) (**Table 1**). Studies have shown that subtypes of EML4-ALK fusion also directly affect the effectiveness of ALK inhibitors. One of the reasons may be that the structural stability of the subtypes is different (24). According to the mechanism by which ALK rearrangement works (25), only EML4 (exon6) - ALK (exon20) fusion could be carcinogenic to our patient. EML4 (exon6) -ALK (exon20) is called variant 3a/b, accounting for 29% of the lung cancers that contain the fusion form of EML4-ALK, and such fusion is more common in East Asians (26–28). In previous study, variant 3a/b is insensitive to ALK inhibitors in NSCLCs (24, 29). Fortunately, the present case definitely benefited from ALK inhibitors. Whether this curative effect in pancreatic cancer has commonality remains to be confirmed by the accumulation of related case libraries, and the difference in the curative effect of the same ALK fusion variant between different tumor species on ALK inhibitors also needs to be further explored.

As with other cancers positive for ALK rearrangement, ALK inhibitors, including crizotinib, ceritinib, alectinib and lorlatinib, can have a good therapeutic effect on ALK rearrangement-positive pancreatic cancer (23, 30, 31).In our dataset, six patients received ALK inhibitors and 5 out of 6 patients were given crizotinib and 5 out of 6 patients received alectinib as the front-line targeted therapy. The best overall response (according to RECIST v.1.1) of these patients after taking ALK inhibitors is shown in **Table 1**. The median PFS of crizotinib was 8.5 months (range 2-17 months), similar to that of ALK rearrangement–positive NSCLC (32, 33).

For other genetic changes in the patient, ARID1B gene is a member of the human SWItch/Sucrose NonFermentable (SWI/SNF) chromatin remodeling complex, which mainly plays a role in tumor suppression. KEAP1 gene is mainly involved in the regulation of redox homeostasis. This mutation is related to the proliferation and chemotherapy resistance of pancreatic cancer. The effectiveness of the patient’s subsequent treatment with ALK inhibitors indicates that ALK rearrangement is the main driving factor for the development of the patient’s tumor, and the above two mutant genes only exist as passenger genes.

However, drug resistance is inevitable. The most common mechanism of resistance to crizotinib in NSCLC is the occurrence of secondary ALK mutations (34). In addition, crizotinib has poor intracranial efficacy due to poor blood-brain barrier (BBB) penetration. In NSCLC, the occurrence of brain metastases (BMs) leads to resistance to crizotinib in up to 60% of patients (35, 36). Our present case experienced BMs without progression in extracranial lesion indicated that the mechanism of resistance to crizotinib was due to the poor penetration of BBB. Alectinib is a potent second generation of ALK inhibitors with high BBB penetration. Its efficacy for BMs has been assessed in phase I-III clinical trials (34). For patients with measurable intracranial disease, the intracranial overall response rate (ORR) was 52%-64%, duration of response (DOR) was about 10 months. Alectinib can reduce the risk of central nervous system (CNS) progression relative to crizotinib by 49% and 81% in patients with or without baseline CNS metastases, respectively (34). However, the patient’s brain metastasis progressed in April 2021, suggesting that there may be resistance to alectinib. At this time, on the one hand, we performed ctDNA testing on the patient, but it has not yet prompted the basis for medication. On the other hand, if the brain metastases continue to progress, we can switch to the third-generation ALK inhibitor lorlatinib, which can inhibit a variety of known ALK resistance mutations and has high blood-brain barrier permeability (37).

Pancreatic cancer usually metastasizes to liver (76%), followed by lung (19.9%), distant lymph nodes (9.4%) and bone (6.8%) (38). Brain metastases are extremely rare (0.6%) (39). This is due to the fact that most patients do not survive long enough to experience the clinical manifestations of brain metastasis. It is also possible that brain imaging studies may not be routinely performed in pancreatic cancer patients without neurological symptoms. It is speculated that brain metastases will be observed with increasing frequency due to the improved prognosis of PDAC patients. Lung metastases might be one of the risk factors for development of brain metastasis in patients with pancreatic cancer (40). This suggest that brain imaging study may be necessary for patients living long and with lung metastases.

ALK rearrangement is extremely rare in pancreatic cancer. BMs are also rare in pancreatic cancer. This is, to our knowledge, the first report of BMs from EML4-ALK fusion pancreatic cancer when treated with crizotinib. To sum up, the present case revealed that ALK inhibitors demonstrate remarkable response in metastatic pancreatic cancer with ALK rearrangement–positive and KRAS-wild after resistance to prior therapy, showing the advantages of precision medicine. What’s more, recently published real-world study suggested that molecular profiling and treatment with molecularly matched therapies are not only feasible, but also significantly improve survival outcomes for a subgroup of patients with pancreatic cancer compared with those for patients who received non-molecularly matched therapies (23). Our present case also suggested that tumor-based molecular profiling for patients with pancreatic cancer should be routinely performed, especially for those who resistant to the standard chemotherapy and still in good performance status. In addition, the incidence of BMs of pancreatic adenocarcinoma is expected to increase due to prolonged survival from improved treatments. Therefore, awareness of brain involvement is necessary when neurological disorder is suspected in patients with advanced pancreatic cancer.

## Data Availability Statement

The original contributions presented in the study are included in the article/**Supplementary Material**. Further inquiries can be directed to the corresponding authors.

## Ethics Statement

The studies involving human participants were reviewed and approved by Departments of Ethics Committee, National Cancer Center/National Clinical Research Center for Cancer/Cancer Hospital, Chinese Academy of Medical Sciences and Peking Union Medical College. The patients/participants provided their written informed consent to participate in this study. Written informed consent was obtained from the individual(s) for the publication of any potentially identifiable images or data included in this article.

## Author Contributions

KO: Conceptualization, Formal analysis, Writing - Original Draft, Writing - Review and Editing. XL: Writing - Original Draft, Writing - Review and Editing. WL: Data Curation, Writing - Review and Editing. YY: Visualization, Writing - Review and Editing. JY: Visualization, Funding acquisition. LY: Writing - Review and Editing, Project administration. All authors contributed to the article and approved the submitted version.

## Funding

This work was supported by the Non-profit Central Research Institute Fund of Chinese Academy of Medical Sciences, 2019PT310026.

## Conflict of Interest

The authors declare that the research was conducted in the absence of any commercial or financial relationships that could be construed as a potential conflict of interest.

## Publisher’s Note

All claims expressed in this article are solely those of the authors and do not necessarily represent those of their affiliated organizations, or those of the publisher, the editors and the reviewers. Any product that may be evaluated in this article, or claim that may be made by its manufacturer, is not guaranteed or endorsed by the publisher.
