# Supplementary figures and images for: ALK Rearrangement–Positive Pancreatic Cancer with Brain Metastasis Has Remarkable Response to ALK Inhibitors: A Case Report
Source: Front Oncol. 2021 Sep 6;11:724815. doi: 10.3389/fonc.2021.724815 (PMC8456297; doi:10.3389/fonc.2021.724815)

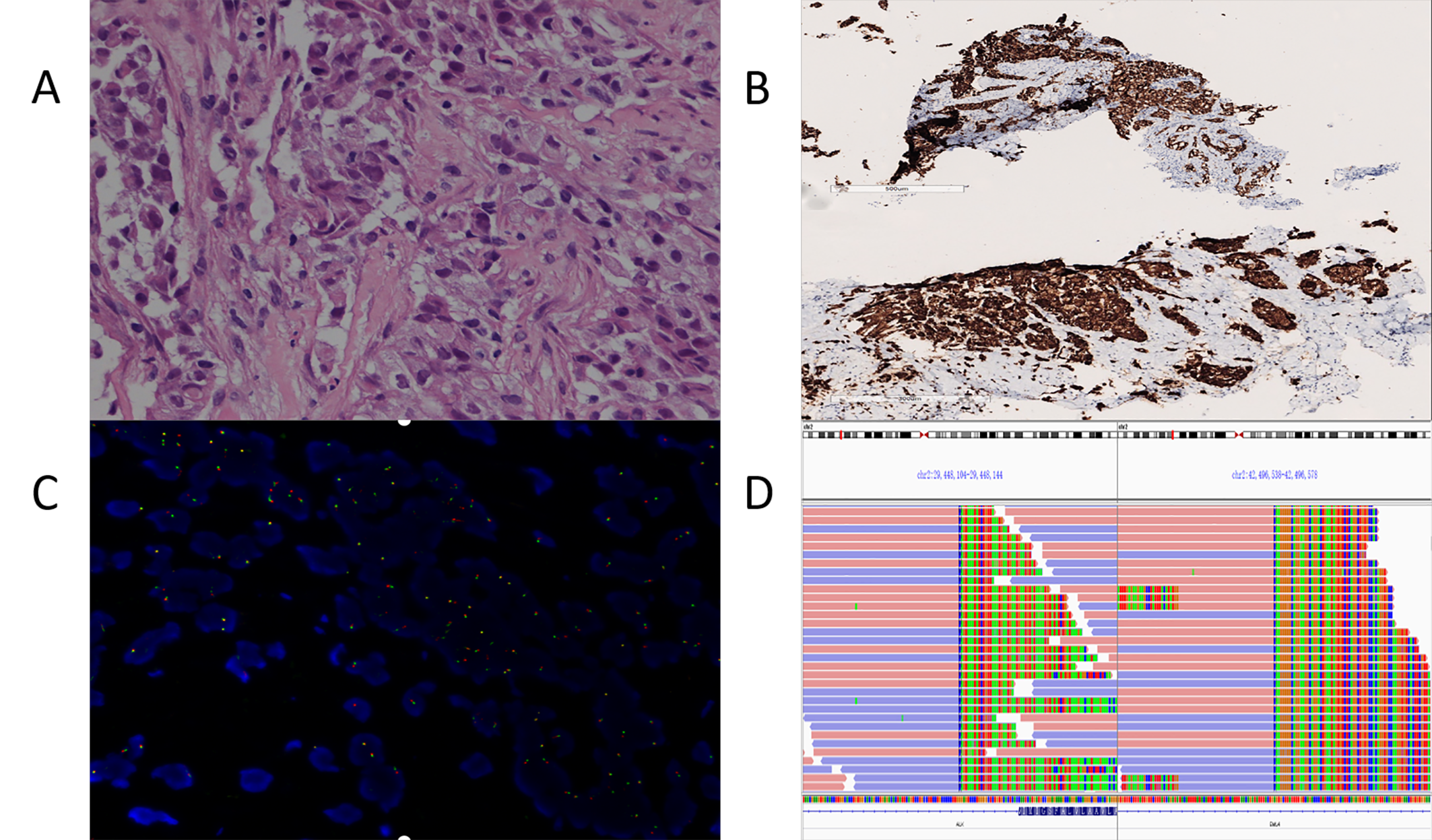

Supplement: Supplementary file 1 [file Image_1.tif]
